# Detection and Significance of CD4+CD25+CD127dim Regulatory T Cells in Individuals with Severe Aplastic Anemia

**DOI:** 10.4274/tjh.2013.0410

**Published:** 2015-08-01

**Authors:** Weiwei Qi, Yue Ren, Rong Fu, Huaquan Wang, Chunyan Liu, Zonghong Shao

**Affiliations:** 1 General Hospital of Tianjin Medical University, Department of Hematology, Tianjin, China

**Keywords:** Severe aplastic anemia, Regulatory T cell, Bone marrow failure

## Abstract

**Objective:**

To investigate the relationship between CD4+CD25+CD127dim regulatory T cells (Tregs) and immune imbalance in acquired severe aplastic anemia (SAA).

**Materials and Methods:**

The quantity of CD4+CD25+CD127dim Tregs in 44 SAA patients and 23 normal controls was measured by flow cytometry. Correlations between Tregs and T cell subsets, dendritic cell (DC) subsets, granulocyte counts, and percentage of reticulocytes (RET%) were analyzed.

**Results:**

The percentage of CD4+CD25+CD127dim Tregs in peripheral blood lymphocytes (PBLs) of untreated patients was lower than in recovery patients and normal controls (0.83±0.44% vs. 2.91±1.24% and 2.18±0.55%, respectively, p<0.05). The percentage of CD4+CD25+CD127dim Tregs in CD4+ T lymphocytes of recovery patients was higher than that of untreated patients and normal controls (9.39±3.51% vs. 7.61±5.3% and 6.83±1.4%, respectively, p<0.05). The percentage of CD4+ T lymphocytes in PBLs of untreated patients was lower than in recovery patients and normal controls (13.55±7.37% vs. 31.82±8.43% and 32.12±5.88%, respectively, p<0.05). T cell subset (CD4+/CD8+ ratio) was 0.41±0.24 in untreated patients, which was lower than in recovery patients (1.2±0.4) and normal controls (1.11±0.23) (p<0.05). DC subset (myeloid DC/plasmacytoid DC ratio, DC1/DC2 ratio) was 3.08±0.72 in untreated patients, which was higher than in recovery patients (1.61±0.49) and normal controls (1.39±0.36) (p<0.05). The percentage of CD4+CD25+CD127dim Tregs in PBLs was positively associated with T cell subset (r=0.955, p<0.01) and negatively associated with DC subset (r=-0.765, p<0.01). There were significant positive correlations between CD4+CD25+CD127dim Tregs/PBL and granulocyte counts and RET% (r=0.739 and r=0.749, respectively, p<0.01).

**Conclusion:**

The decrease of CD4+CD25+CD127dim Tregs in SAA patients may cause excessive functioning of T lymphocytes and thus lead to hematopoiesis failure in SAA.

## Abstract (tr)

**Amaç:**

Edinilmiş ağır aplastik anemilerde (AAA) rastlanan CD4+CD25+CD127dim düzenleyici T hücreleri (Tregs) ve bağışıklık bozukluğu arasındaki ilişkiyi araştırmayı amaçladık.

**Gereç ve Yöntemler:**

Kırk dört AAA hastasındaki ve 23 normal kontroldeki CD4+CD25+CD127dim Tregs miktarıları akış sitometrisi kullanarak ölçüldü. Tregs ve T hücrelerinin alt tipleri, dentrik hücrelerin alt tipleri (DH), granülosit sayımları ve retikülosit yüzdeleri (%RET) aralarındaki ilişkiler analiz edildi.

**Bulgular:**

Tedavi edilmemiş hastaların çevresel kan lenfositlerindeki (ÇKL) CD4+CD25+CD127dim Tregs’in yüzdesi iyileşme sürecindeki hastalarla ve normal kontrollerle kıyaslandığında daha düşüktü (sırasıyla %0,83±0,44’e karşı %2,91±1,24 ve %2,18±0,55, p<0,05). İyileşme sürecindeki hastaların CD4+ T lenfositlerindeki CD4+CD25+CD127dim Tregs’in yüzdesi tedavi edilmemiş hastalarla ve normal kontrollerle kıyaslandığında daha yüksekti (sırasıyla %9,39±3,51’e karşı %7,61±5,3 ve %6,83±1,4, p<0,05). Tedavi edilmemiş hastaların CD4+ T lenfositlerin yüzdesi iyileşme sürecindeki hastalarla ve normal kontrollerle kıyaslandığında daha düşüktü (sırasıyla %13,55±7,37’ye karşı %31,82±8,43 ve 32,12±5,88, p<0,05). Tedavi edilmemiş hastalardaki T hücresi alt tipi (CD4+/CD8+ oran) 0,41±0,24 seviyesindeydi; bu oran iyileşme sürecindeki hastalarla (1,2±0,4) ve normal kontrollerle (1,11±0,23) kıyaslandığında daha düşük bir seviyeydi (p<0,05). DH alt dizini (miyeloid DH/plazmasitoid DH oranı, DH1/DH2 oranı) ise tedavi edilmemiş hastalarda 3,08±0,72 seviyesindeydi; bu oran iyileşme sürecindeki hastalarla (1,61±0,49) ve normal kontrollerle kıyaslandığında (1,39±0,36) daha yüksekti (p<0,05). ÇKL’deki CD4+CD25+CD127dim Tregs’in yüzdesi pozitif olarak T hücresi alt kümesiyle (r=0,955, p<0,01) ve negatif olarak da DH alt kümesiyle (r=-0,765, p<0,01) korele etmekteydi. CD4+CD25+CD127dim Tregs/ÇKL ve granülosit değerleri ve %RET (sırasıyla r=0,739, 0,749, p<0,01, p<0,01) arasında önemli pozitif korelasyon saptandı.

**Sonuç:**

AAA hastalarındaki CD4+CD25+CD127dim Tregs’in azalması T lenfositlerde aşırı fonksiyona sebebiyet verebilir ve bu durum AAA’da hematopoetik yetmezliğine yol açabilir.

## INTRODUCTION

Severe aplastic anemia (SAA) is a rare disease characterized by severe pancytopenia and bone marrow failure. Recent studies confirmed that aplastic anemia (AA) is an immune-mediated disease characteristic of hematopoietic cells destroyed by activated T lymphocytes [1,2,3]. Activated suppressor T lymphocytes are markedly increased in SAA patients and suppress normal hematopoiesis in vitro. In addition, suppressive cytokines produced by activated T lymphocytes also play a vital role in the pathogenesis of bone marrow failure. High levels of suppressive cytokines indicate an excessive Th1 type of T cell response. Recent studies have shown that myeloid dendritic cells (DCs), which also expressed high levels of costimulatory molecules (CD86), were increased in AA and were associated with disease severity. This could explain why naive T cells are prone to differentiating into Th1 cells [4]. Furthermore, CD4+CD25+FOXP3+ regulatory T cells (Tregs) were decreased at presentation in almost all patients with AA, and a defect in Tregs might explain the increased autoreactive T cells and the development of an AA phenotype [5].

Tregs are a suppressive subset of T cells, which are essential for maintaining appropriate immune self-tolerance and balance. The main function of Tregs is their powerful capacity to suppress T effector or memory cell activation as well as to regulate self-reactive T cells [6]. Clinical studies have demonstrated a decreased suppressive ability and quantity of Tregs in patients with autoimmune diseases such as systemic lupus erythematosus and multiple sclerosis [7,8]. Previous studies from our group and others suggested that CD4+CD25+ Tregs were decreased in untreated SAA patients compared with recovery patients and normal controls [9,10]. However, CD25 is not a unique Treg marker because it is also present on activated T cells. Recently, studies proposed that low expression of CD127 coexpressed with Foxp3 and CD25, particularly in humans, could be used to distinguish Tregs from activated CD4+ T cells [11].

In this study, we focused on detecting the quantity of Tregs in SAA, which is considered an autoimmune disease, and we analyzed the relationship between Tregs and the severity of disease as well as T cell subset and DC subset.

## MATERIALS AND METHODS

### Study Subjects

A total of 44 patients with SAA were enrolled in this study, including 25 untreated cases and 19 cases in remission after immunosuppressive therapy. All were inpatients of the Hematology Department of Tianjin Medical University General Hospital, China, from January 2013 to October 2013. The diagnosis of SAA was based on International AA Study Group criteria [2]. Patients were excluded if they had congenital AA or other autoimmune diseases. Paroxysmal nocturnal hemoglobinuria (PNH) clone and bone marrow cytogenetic studies were performed for all patients. The patients’ features are shown in Tables 1, 2, and 3. Twenty-three healthy controls with a median age of 33 years (range: 21-50) were also enrolled in this study, including 6 males and 17 females. The study was approved by the Ethics Committee of Tianjin Medical University, China. Informed written consent was obtained from all patients or their parents in accordance with the Declaration of Helsinki.

### Monoclonal Antibodies

CD4-PE, CD25-FITC, CD127-APC, FITC-lineage-cocktail (CD3, CD14, CD16, CD19, CD20, CD56), HLA-DR-PerCP, CD123-PE, CD11c-APC, CD3-PE-Cy5, CD4-FITC, CD8-PE, mouse isotype controls, and lysis solution were purchased from Becton Dickinson (Franklin Lakes, NJ, USA).

### Measurement of Quantities of Tregs from Peripheral Blood

CD4+CD25+CD127dim Tregs were identified by single-platform 3-color flow cytometric analysis. Fresh blood samples of 200 µL processed by ethylenediaminetetraacetic acid were separated into 2 TruCount tubes (Becton Dickinson) and stained with mouse IgG1-PE, mouse IgG1-FITC, and mouse IgG1-APC as a negative control, or with CD4-PE, CD25-FITC, and CD127-APC. After incubation in the dark for 20 min at 4 °C, cells were incubated with 1 mL of lysis solution (Becton Dickinson) for 10 min at room temperature and washed 3 times with phosphate buffered saline. Each specimen was acquired on a FACS-Calibur flow cytometer and analyzed using CellQuest 3.1 software (Becton Dickinson).

### Measurement of T Cell Subsets and Dendritic Cell Subsets in Peripheral Blood

CD3+CD4+ and CD3+CD8+ T lymphocytes were stained with CD3-PE-Cy5, CD4-FITC, and CD8-PE. Myeloid DC (DC1) was identified as lineage(-)HLA-DR(+)CD11c(+), while plasmacytoid DC (DC2) was identified as lineage(-)HLA-DR(+)CD123(+). DC1 and DC2 were stained with FITC-lineage-cocktail, HLA-DR-PerCP, CD123-PE, and CD11c-APC. Further staining followed the protocol described above.

### Statistical Analysis

Statistical analysis was performed by parametric t-test. Correlation studies were performed by Spearman analysis. A value of p<0.05 was considered statistically significant.

## RESULTS

### Decreased Numbers of Tregs in Untreated Severe Aplastic Anemia Compared with Healthy Controls

**Figure 1:**
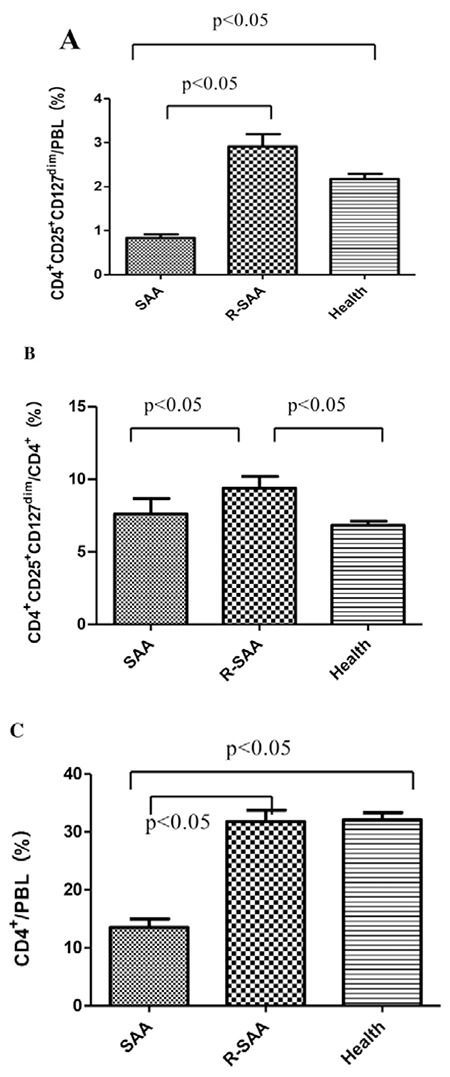
The quantities of Tregs and CD4+ T cells in untreated severe aplastic anemia patients (SAA), recovery patients (R-SAA), and normal controls (Health). A) The percentage of CD4+CD25+CD127dim Tregs in peripheral blood lymphocytes. B) The percentage of CD4+CD25+CD127dim Tregs in CD4+ T cells. C) The percentage of CD4+ T cells in peripheral blood lymphocytes.

The percentage of CD4+CD25+CD127dim Tregs in peripheral blood lymphocytes (PBLs) was 0.83±0.44% in untreated SAA patients, 2.91±1.24% in recovery patients, and 2.18±0.55% in normal controls. The percentage of CD4+CD25+CD127dim Tregs in PBLs in untreated SAA patients was lower than in recovery patients and normal controls (p<0.05), and there was no statistical difference between recovery patients and normal controls (p>0.05). The percentage of CD4+CD25+CD127dim in CD4+ T cells in recovery patients (9.39±3.51%) was higher than in untreated patients (7.61±5.3%) and normal controls (6.83±1.4%) (p<0.05), and there was no statistical difference between recovery patients and normal controls (p>0.05). The percentage of CD4+ T cells in PBLs from untreated patients (13.55±7.37%) was lower than in recovery patients (31.82±8.43%) and normal controls (32.12±5.88%) (p<0.05), while there was no statistical difference between recovery patients and normal controls (p>0.05) (Figure 1).

### T Cell Subsets and Dendritic Cell Subsets Were Altered in Severe Aplastic Anemia Patients

**Figure 2:**
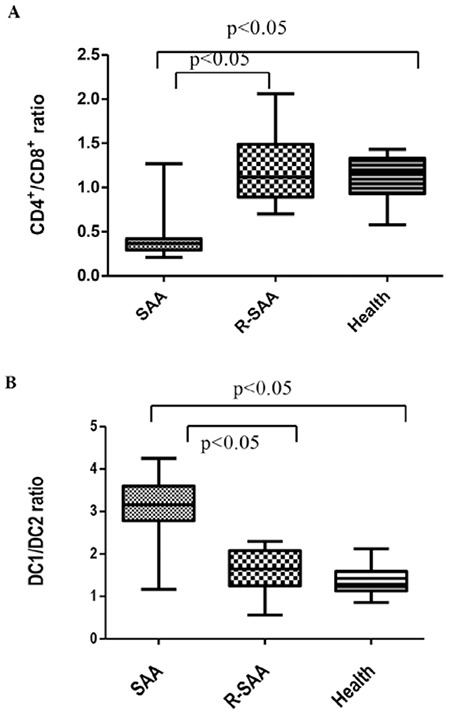
The T cell subsets and dendritic cell subsets in untreated severe aplastic anemia patients (SAA), recovery patients (R-SAA), and normal controls (Health). A) The T cell subsets (CD4+/CD8+ ratios) in the 3 groups. B) The dendritic cell subsets (DC1/DC2 ratios) in the 3 groups.

T cell subset (CD4+/CD8+ ratio) was 0.41±0.24 in untreated SAA patients, which was significantly lower than in recovery patients (1.2±0.4) and normal controls (1.11±0.23) (p<0.05). There was no difference between recovery patients and normal controls (p>0.05). DC subset (myeloid DC/plasmacytoid DC ratio, DC1/DC2 ratio) was 3.08±0.72 in untreated SAA patients, which was significantly higher than in recovery patients (1.61±0.49) and normal controls (1.39±0.36) (p<0.05). There was no difference between recovery patients and normal controls (p>0.05) (Figure 2).

### Correlations between CD4+CD25+CD127dim Tregs and T Cell Subsets, Dendritic Cell Subsets, Granulocytes, and RET% in Severe Aplastic Anemia Patients

**Figure 3:**
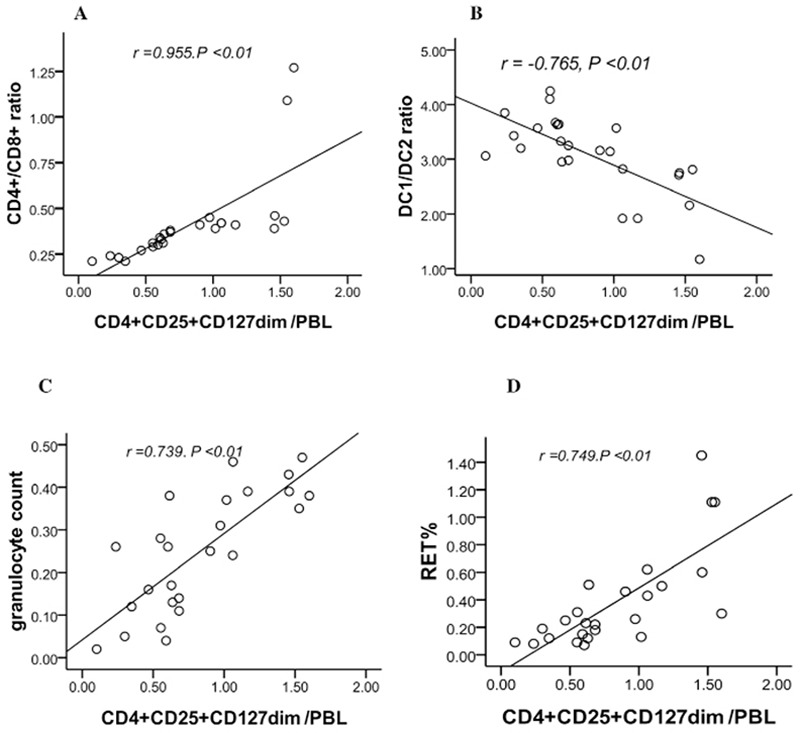
The correlations between CD4+CD25+CD127dim Tregs and T cell subsets, dendritic cell subsets, granulocytes, and RET% in severe aplastic anemia patients. A) The correlation between the percentage of CD4+CD25+CD127dim Tregs in peripheral blood lymphocytes (PBLs) (CD4+CD25+CD127dim/PBL) and T cells subsets (CD4+/CD8+ ratios). B) The correlation between CD4+CD25+CD127dim/PBL and dendritic cell subsets (DC1/DC2 ratios). C) The correlation between CD4+CD25+CD127dim/PBL and granulocyte count. D) The correlation between CD4+CD25+CD127dim/PBL and RET%.

The percentage of CD4+CD25+CD127dim Tregs in PBLs (CD4+CD25+CD127dim Tregs/PBL) was significantly and positively associated with T cell subset (r=0.955, p<0.01). However, CD4+CD25+CD127dim Tregs/PBL was negatively associated with DC subset (r=-0.765, p<0.01). There were positive correlations between CD4+CD25+CD127dim Tregs/PBL and granulocyte counts and RET% (r=0.739 and r=0.749, respectively, p<0.01). There was no significant correlation between CD4+CD25+CD127dim Tregs/PBL and hemoglobin and platelets (Figure 3).

## DISCUSSION

Tregs are considered to play a key role in the mechanisms of peripheral immune tolerance. Tregs use various mechanisms such as cell-to-cell contact and secretion of soluble factors to suppress immune responses. There are several specific mechanisms included, such as the release of inhibitory cytokines and immunosuppressive adenosine; inhibition of IL-2 secretion; perforin- or granzyme-dependent cytolysis of effector cells; and down-regulation of antigen present cell (APC) function via costimulation with CTLA-4 [12]. There are 2 types of Tregs: natural Tregs (nTregs), which develop in the thymus, and induced Tregs (iTregs), which differentiate from naive CD4+ T cells in the periphery. Foxp3 is the most specific marker of nTregs and is critical for the development and maintenance of the suppressive function of Tregs. However, Foxp3 is expressed in the nucleus of Tregs, and therefore it cannot be used to identify nTregs during isolating and purification. It was recently shown that CD127, a specific Treg surface marker, is inversely correlated with Foxp3 expression and the suppressive capacity of nTregs [13]. In the current study, we used the characteristics of constitutively high expression of CD25 and diminished expression of CD127 to discriminate Tregs from conventional CD4+ T cells.

Cellular immunity is abnormal in SAA patients. Numerous studies have shown that both the quantity and the function of myeloid DCs are increased, that Th1 cells are excessively activated along with excessive secretion of Th1-type cytokines, that the ratio of CD4+/CD8+ T lymphocytes is decreased, and that cytotoxic T cells are over activated [1,4]. Currently, the consensus is that acquired AA is an autoimmune disease. AA pathogenesis is thought to involve damage to hematopoietic stem/progenitor cells by autoreactive T lymphocytes activated by Th1 cells. An important question is why these autoreactive T cells are not suppressed and removed by self-tolerance. Furthermore, what is the influence of Tregs on the complex pathogenesis of AA? Our results demonstrated that the percentage of CD4+CD25+CD127dim Tregs in the peripheral blood was decreased in SAA patients. Furthermore, the percentage of CD4+CD25+CD127dim Tregs increased and was restored to normal levels in the patients after intensive immunosuppressive therapy. Additionally, in SAA patients with a more serious condition, we noted that their granulocyte counts and RET% were markedly decreased, DC1 and DC1/DC2 ratios were increased, CD4+/CD8+ ratios were decreased, and Tregs were decreased. After statistical analysis, we concluded that the percentage of CD4+CD25+CD127dim Tregs in PBLs was negatively associated with DC subset and positively associated with T cell subset, granulocyte counts, and RET%. These results suggested that Tregs were involved in the pathogenesis of SAA and inversely associated with the severity of disease. We postulate that decreased numbers of Tregs fail to suppress the function of DCs and activated T cells, which impair and damage hematopoietic cells in the bone marrow. Our results also indicated that the antithymocyte globulin (ATG) plus cyclosporine therapeutic regimen induced the proliferation of Tregs. This was consistent with a previous study using in vitro cultures of normal human peripheral blood mononuclear cells treated with low-dose rabbit ATG that markedly expanded functional Tregs [14]. These findings indicated that Tregs might be protective against SAA. Furthermore, CD4+CD25+CD127dim Tregs could be used as a biomarker to determine the curative effect of treatments and evaluate the prognosis of SAA. During SAA patient recovery, the quantity of CD4+CD25+CD127dim Tregs increased gradually, suggesting a better prognosis. It is essential that the function of Tregs be studied and analysis of Tregs at different stages of SAA be conducted in the future.

Recent studies have explored new methods of expanding or inducing de novo generation of Tregs, which could provide a new approach for the treatment of autoimmune diseases. Cao et al. showed that Tregs from patients with autoimmune diseases could be purified, activated, and expanded similarly to those from healthy donors. Additionally, ex vivo expanded Tregs showed enhanced suppressive functions compared with Tregs freshly purified from the same patients [15]. Studies using murine models confirmed that Treg adoptive transfer is feasible and efficient for treating autoimmune disease [16]. Taken together, these studies suggest that new therapeutic strategies based on Tregs for the treatment of SAA might be beneficial.

## Figures and Tables

**Table 1:**
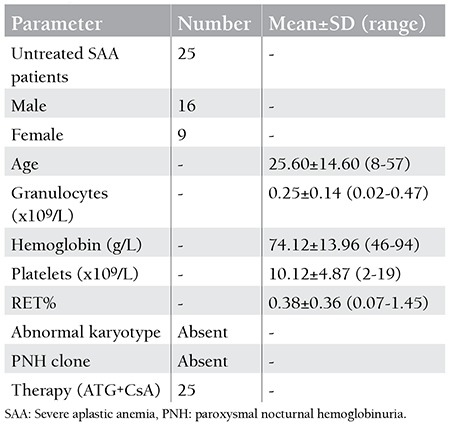
Characteristics of untreated severe aplastic anemia patients.

**Table 2:**
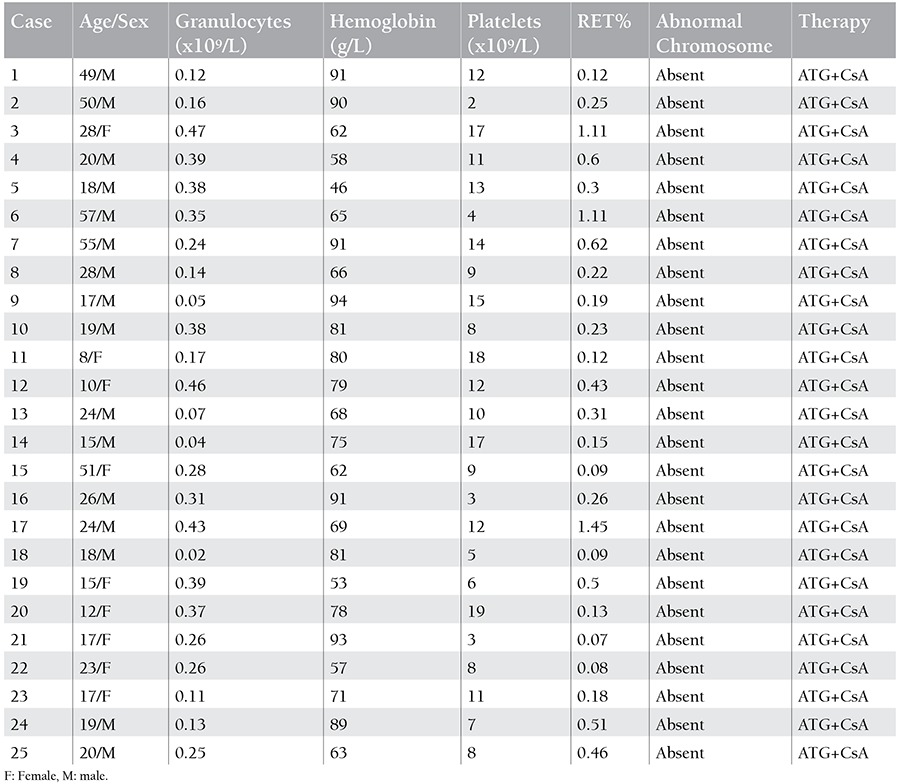
Characteristics of untreated severe aplastic anemia patients.

**Table 3:**
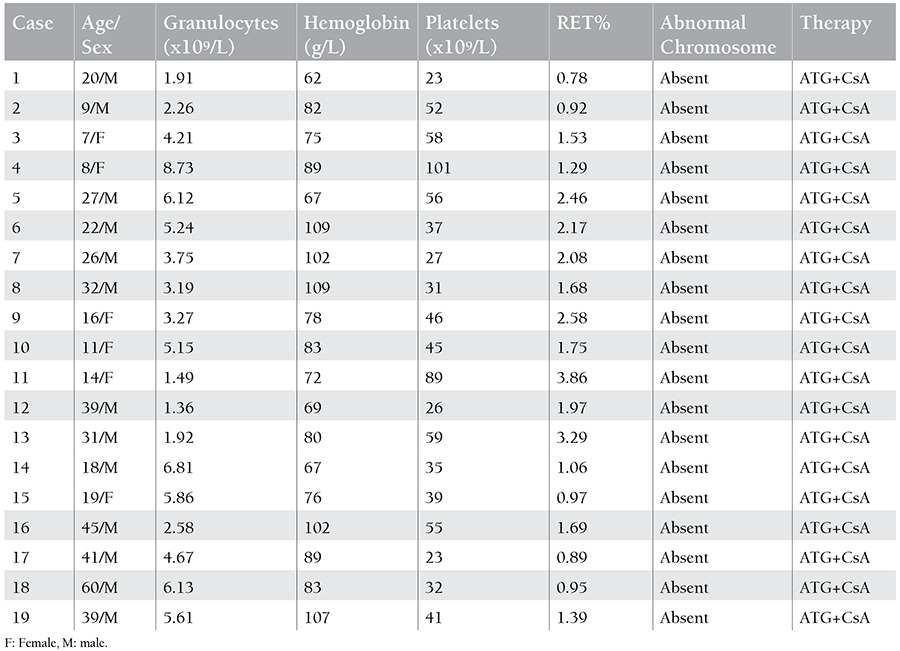
Characteristics of recovery severe aplastic anemia patients.
